# Deciphering the introduction and transmission of SARS-CoV-2 in the Colombian Amazon Basin

**DOI:** 10.1371/journal.pntd.0009327

**Published:** 2021-04-15

**Authors:** Nathalia Ballesteros, Marina Muñoz, Luz Helena Patiño, Carolina Hernández, Felipe González-Casabianca, Iván Carroll, Mauricio Santos-Vega, Jaime Cascante, Andrés Angel, Alejandro Feged-Rivadeneira, Mónica Palma-Cuero, Carolina Flórez, Sergio Gomez, Adriana van de Guchte, Zenab Khan, Jayeeta Dutta, Ajay Obla, Hala Alejel Alshammary, Ana S. Gonzalez-Reiche, Matthew M. Hernandez, Emilia Mia Sordillo, Viviana Simon, Harm van Bakel, Alberto E. Paniz-Mondolfi, Juan David Ramírez

**Affiliations:** 1 Centro de Investigaciones en Microbiología y Biotecnología-UR (CIMBIUR), Facultad de Ciencias Naturales, Universidad del Rosario, Bogotá, Colombia; 2 Gestión y desarrollo urbanos, Facultad de Estudios Internacionales, Políticos y Urbanos, Universidad del Rosario, Bogotá, Colombia; 3 Facultad de Ingeniería, Universidad de Los Andes, Bogotá, Colombia; 4 Grupo de biología matemática y computacional, Departamento de Ingeniería Biomédica, Universidad de los Andes, Bogotá, Colombia; 5 Departamento de Matemáticas, Universidad de Los Andes, Bogotá, Colombia; 6 Laboratorio de Salud Púbica Departamental de Amazonas, Leticia, Colombia; 7 Instituto Nacional de Salud, Bogotá, Colombia; 8 Department of Genetics and Genomic Sciences, Icahn School of Medicine at Mount Sinai, New York, New York, United States of America; 9 Department of Microbiology, Icahn School of Medicine at Mount Sinai, New York, New York, United States of America; 10 Department of Pathology, Molecular and Cell Based Medicine, Icahn School of Medicine at Mount Sinai, New York, New York, United States of America; 11 The Global Health and Emerging Pathogens Institute, Icahn School of Medicine at Mount Sinai, New York, New York, United States of America; 12 Division of Infectious Diseases, Department of Medicine, Icahn School of Medicine at Mount Sinai, New York, New York, United States of America; University of Reunion Island, RÉUNION

## Abstract

**Background:**

The SARS-CoV-2 pandemic has forced health authorities across the world to take important decisions to curtail its spread. Genomic epidemiology has emerged as a valuable tool to understand introductions and spread of the virus in a specific geographic location.

**Methodology/Principal findings:**

Here, we report the sequences of 59 SARS-CoV-2 samples from inhabitants of the Colombian Amazonas department. The viral genomes were distributed in two robust clusters within the distinct GISAID clades GH and G. Spatial-temporal analyses revealed two independent introductions of SARS-CoV-2 in the region, one around April 1, 2020 associated with a local transmission, and one around April 2, 2020 associated with other South American genomes (Uruguay and Brazil). We also identified ten lineages circulating in the Amazonas department including the P.1 variant of concern (VOC).

**Conclusions/Significance:**

This study represents the first genomic epidemiology investigation of SARS-CoV-2 in one of the territories with the highest report of indigenous communities of the country. Such findings are essential to decipher viral transmission, inform on global spread and to direct implementation of infection prevention and control measures for these vulnerable populations, especially, due to the recent circulation of one of the variants of concern (P.1) associated with major transmissibility and possible reinfections.

## Author summary

SARS-CoV-2 has dramatically impacted Amerindian native communities across South America, particularly in the Amazonian basin. In order to unveil the introduction and initial spread of this pandemic virus into this region, we conducted a genomic epidemiology study where we sequenced 59 genomes from cases in the Amazonas department of Colombia. Our results showed two independent introductions of the virus into the department, one of these associated with asymptomatic cases. This represents the first genomic epidemiology study focused on the Colombian Amazonas department where a great amount of native Amerindian indigenous communities inhabits. Our results provide insights of the transmission dynamic in this region and reported relevant information to pursue strategies to mitigate the spread of the virus in the Amazon population which currently are facing new risks due to the circulating new variant of concern P.1.

## Introduction

The emergence of the Severe Acute Respiratory Syndrome Coronavirus 2 (SARS-CoV-2) has created a worldwide crisis, derailing public health systems and the provisioning of healthcare. After the first reports of severe respiratory disease caused by this virus in Wuhan, China in December 2019, SARS-CoV-2 spread rapidly and the World Health Organization declared Coronavirus Disease 2019 (COVID-19) a pandemic on March 11, 2020 [1]. As of February 28, 2021, approximately 114.2 million cases and 2.5 million deaths have been reported globally [2]. In South America, the first case was reported on February 25, 2020, in Sao Paulo, Brazil. On March 20, 2020, confirmed cases were reported in all Latin American countries, and as of February 28, 2021, 50.5 million infections and 1.2 million deaths were reported in the region [3].

The Amazon region has not been spared by the COVID-19 pandemic. The first case was confirmed on March 13, 2020, in Manaus [4], which serves as a major departure point from Brazil to the surrounding Amazon. After the first reported death on March 24, 2020, confirmed cases in the area increased exponentially from 67 to 315,966 (March 26 to February 28, 2021) [4]. The extensive spread has threatened people in the 690 indigenous territories that comprise the Amazon in Brazil [5,6]. Indeed, recent seroprevalence studies have demonstrated spread of the disease in Manaus [7]. Nonetheless, transmission events and evolution of SARS-CoV-2 among these inhabitants are poorly understood [8].

Inhabitants of the Colombian Amazon (Amazonas department) have similarly been overwhelmingly affected by COVID-19. Overall, as of February 28, 2021, Colombia has reported 2,251,690 confirmed cases and 59,766 deaths [9]. Specifically, the department of Amazonas has reported 5,093 cases and 177 deaths, with the majority of cases (94.3%) from its capital, Leticia [9]. In the Colombian Amazon basin, there are approximately 26 indigenous ethnic groups comprised of 47,000 individuals. As of November 2020, SARS-CoV-2 cases and deaths have been reported among the Arawak, Tikuna, and Tukano ethnic groups. These cases are thought to be the result of directional spread from the bordering Tabatinga, Brazil, which had reported 2,999 infections and 111 deaths as of February 28, 2021 [4]. However, SARS-CoV-2 transmission dynamics are currently poorly understood within these communities which are uniquely vulnerable to infectious disease [10–13].

In fact, the COVID-19 epidemic in the Amazonas department of Colombia is characterized by two peaks of exponential growth incidence curves, the first during May 2020 with the highest number of cases per capita in the country, followed by continuous decreased in the number of the cases in the following months. However, at the beginning of 2021 the cases and incidence started to increase, establishing the second peak, currently being one of the Colombian departments with the highest incidence per capita in the country. These observed fluctuations in the incidence in this region such as in other countries is a consequence of the changes in the viral dynamics and the emergence of new variants of concern (VOCs) [14]. One of these VOCs is the lineage P.1, first detected on January 2, 2021 in Manaus in four travelers returning from the Amazonas state, Brazil [15], and in Colombia on January 29, 2021 in Leticia, Amazonas in a woman who crossed the border between Brazil and Colombia to attend to medical consultation in the hospital of this city [16]. The mutations defining the P.1 lineage include 10 synapomorphic mutations in the Spike protein and have been associated with possible reinfection cases, increase in the viral load and a major transmissibility [15,17]. Nevertheless, the possibility of reinfections related to this variant remain unclear and more studies are needed to evaluate the impact of the pandemic dynamics.

Genomic epidemiology studies have enabled not only the characterization and spread of the VOCs and other viral lineages, but also has been widely implemented to decipher regional transmission dynamics [18], introduction events, and the high-resolution reconstruction of transmission patterns of SARS-CoV-2 in several countries, including China [19], the US (Washington [20], California [21], New York [22]), Chile [23], Colombia [24], Brazil [25], Panama [26], among others.

Although we previously identified at least 9 introductions in Colombia [24], there have been no reports on SARS-CoV-2 transmission in the Colombian Amazon, an area with limited access to health services rendering its indigenous inhabitants particularly vulnerable. To address this, we sequenced 59 viruses from specimens collected principally in Leticia (Amazonas department) and conducted phylogenetic and evolutionary analysis against a set of 9,653 publicly available genomes representing the global background diversity, to decipher the clades circulating in this region. Herein, we identified two clusters with potential independent introductions of SARS-CoV-2 in the Colombian Amazon basin. Together, these findings shed light on SARS-CoV-2 transmission dynamics in indigenous territories and can direct further implementation of infection control measures in the region, specially, with the recent increase of cases and the presence of the VOC P.1.

## Methods

### Ethics statement

The Colombian National Institute of Health (INS) is designated as the reference laboratory in Colombia. When a public health emergency occurs, the INS is authorized under national law 9-1979, decrees 786-1990 and 2323-2006, to use biospecimens and associated epidemiological information without informed consent, including the anonymous disclosure of results. This study was performed following the Declaration of Helsinki and its later amendments, and all patient data was anonymized to minimize risk to participants.

### Study area

The Amazonas Department is situated between 00° 07’08” north latitude and 04° 13’ 19” south latitude; and 69° 39’ 41” and 74° 23’ 21” west longitude. Occupying 109,665 km^2^ and totaling 42% of the geographic area of Colombia, it is at once the largest Department in the country and the least populated. Amazonas is part of the Amazon basin, the largest forest region in the world, shared by Venezuela, Brazil, Colombia, Ecuador, Peru, Guyana, Suriname, and Bolivia. The Department includes two municipalities (Leticia and Puerto Nariño), and nine inhabited areas that do not belong to any municipality, (El Encanto, La Chorrera, La Pedrera, La Victoria, Miriti-Parana, Puerto Alegria, Puerto Arica, Puerto Santander, and Tarapacá). Most of the Departments’ geographical landscape is covered by endemic dense, tropical rain forest with a total population of 79,020 inhabitants as reported by DANE (https://www.minsalud.gov.co/sites/rid/Lists/BibliotecaDigital/Forms/DispForm.aspx?ID=2044).

Leticia is the capital of the department and the southernmost city in Colombia bordering with Brazil, and Peru. It shares an open border in conurbation with its neighboring city of Tabatinga in Brazil and is not accessible by road, being only reachable by air or river ways [27]. Its population is heterogeneous formed by domestic and Brazilian immigrants, as well as indigenous people from different ethnic groups from nearby locations. More recently, the rise of tourism and increase in rotating population has transformed the city into a multicultural hub.

### Epidemiological data

The incidence of SARS-CoV-2 in the Colombian Amazonas department was analyzed from the data retrieved from the COVID-19 positive cases dataset reported by the National Institute of Health, Colombia (https://www.ins.gov.co/Noticias/Paginas/Coronavirus.aspx) from April 2020 to January 2021, and the population census reported by the National Administrative Department of Statistics, Colombia (https://www.dane.gov.co/). Similarly, for calculating the incidence of the whole country the complete information of all departments including Amazonas was retrieved from the dataset mentioned above, from the first positive case reported until January 2021. Lastly, the map of incidence by Colombian departments was constructed in QGIS (QGIS Geographic Information System, Open-Source Geospatial Foundation Project, http://qgis.osgeo.org). We also estimated the time varying reproductive number Rt using EpiNow2 that currently estimates that following the best practice for estimate Rt accounting for delay report between symptom onset and confirmation by diagnosis and directly accounting for other biology delays since infection and symptom onset [28–30].

### Collection of nasopharyngeal swab samples that tested positive for SARS-CoV-2

Individuals meeting criteria established by the Ministry of Health were screened for SARS-CoV-2 infection at two hospitals in Leticia, Amazonas between April 25, 2020, and May 5, 2020. The Colombian National Institute of Health is the national reference center for molecular detection of SARS-CoV-2 in the country. This Institute authorized the Universidad del Rosario, Bogotá on March 28, 2020 to perform SARS-CoV-2 diagnostic testing in an attempt to increase diagnostic capacity nationwide. Nasopharyngeal swabs in viral transport media (NP-VTM) collected from suspected patients were submitted to Universidad del Rosario for diagnostic testing. A total of 59 specimens were included in the study.

### Diagnostic testing for SARS-CoV-2 and preparation of total RNA for sequencing

Molecular detection of SARS-CoV-2 in clinical NP-VTM specimens was performed using the Berlin-Charité real-time RT-PCR assay previously described [31]. Briefly, the assay targets the region of the ORF1ab (RdRp) gene, which is unique to SARS-CoV-2 and a conserved region in the E gene for panSarsbecovirus including SARS-CoV-2. Amplification of both targets was required to call a specimen positive.

Total RNA was extracted from 59 clinical specimens that tested positive for SARS-CoV-2 by lysis of 280 μL of NP-VTM with AVL buffer. Viral RNA was extracted using the QIAamp Viral RNA Minikit (QIAGEN, cat. 52904), per the manufacturer’s instruction.

### Whole-genome amplification and sequencing

Sample preparation for sequencing was done using whole-genome amplification based on the Artic Consortium protocol (https://artic.network/ncov-2019) [32], with modifications and a custom tiling primer set as previously reported [22]. Amplicon libraries were prepared with the Nextera XT kit according to the manufacturer instructions and were sequenced on the Illumina MiSeq platform in paired-end format (2x 150bp reads).

### Genome assembly and data retrieval

A total of 59 viral genomes obtained from NP swabs from patients in Leticia (N = 57), Puerto Nariño (N = 1), and Tarapacá (N = 1) were generated and used to reconstruct SARS-CoV-2 genomes using a custom reference-based assembly pipeline (https://github.com/mjsull/COVID_pipe), as previously described [22]. To these genomes were added 36 genomes publicly available in the GISAID EpiCoV database from Amazonas, Colombia, for a total dataset of 95 sequences.

Additionally, a SARS-CoV-2 reference dataset was compiled from publicly available genomes downloaded from GISAID for comparative genomic analyses spanning the date of the first genome released in the GISAID database to the last collection date of the Amazonas department genomes included in this study (January 30, 2021). This dataset initially included all the entries from all the regions including North America, South America, Europe, Africa, Asia, and Oceania in the temporal window mentioned above. Then, only the sequences with a maximum proportion of 0.2 Ns were selected and finally, for all the regions except for South America, they were filtered based in the GISAID clade, country, and Pangolin lineage representability including members of all the lineages per country. The filtered selection was complemented with the genomes from South America that represent all the diversity of SARS-CoV-2 circulating in the region; after sequence alignment (described in the following section), all the South American sequences redundant (with 100% of identity) were excluded from the subsequent analyses. As a result, we utilized 9,653 final global background genome sequences in addition to the 59 genomes from this study.

### Phylogenetic analyses

The 59 SARS-CoV-2 genomes had >99% completeness and were aligned with MAFFT v7.455 using the FFT-NS-2 algorithm and default parameter settings [33]. Untranslated regions were subsequently trimmed in Unipro UGENE v.36.0 [34]. Maximum likelihood (ML) phylogenies were inferred using the IQ-TREE multicore version 2.0.3 [35] using GTR as the best substitution model. The robustness of the nodes was evaluated using the Bootstrap method (BT, with 1,000 replicates). Clade information of publicly available genomes was compared across tree topology and then was used as reference to conduct GISAID clade assignment.

The alignment generated for the complete dataset (9,712 genomes; including 59 from this study) was time-scaled using a maximum-likelihood phylodynamic analysis in TreeTime **(S1 Table)** [35]. For this, the initial ML tree topology obtained from IQ-TREE, and the Collection Dates as set of date constraints (tip dates) were considered as inputs. During the time-scaled analysis a fixed clock rate of 0.8×10^-3^ (SD = 0.4×10^-3^), in agreement with rate values estimated by others [36], a strict clock (SC) under a skyline coalescent tree prior, and a step of root to minimize residuals on a root-to-tip were defined. The TreeTime analyses were run for a total of 6 iterations. Marginal date estimates of ancestral states were inferred with 95% confidence intervals (95% CI). The tree was graphically represented using Microreact [37].

### Clusters identification

The clusters in which the 59 Amazonas genomes were included were pruned to identify the similarity between the genomes of each one. To assess the dissimilarity, a distance matrix was calculated and graphically represented by a heatmap with the Pearson distance measurement method. The criteria to select the clusters were: 1) the genomes belonged to the same geographical point; 2) it included the largest number of related Amazonas isolates; 3) a distance matrix was performed to evaluate the dissimilarity between the cluster and the remaining genomes in the same clade (the distance between the cluster members have to be nearest to zero and with the outlier genomes have to be further to zero).

### Estimation of potential introduction date

To verify the inferred time to the most recent common ancestor (tMCRA), it was pruned the clade containing the clusters (C1 and C2), which together with all the remaining Amazonas and Colombian genomes of the global background (9,653 genomes), and a representability selection of genomes from Brazilian, South American, and Other regions (the first genome per lineage per category) was established as a subsampled dataset with 1441 genomes (**S2 Table**). Phylogeographic relationships were comprehensively analyzed from the SNPs alignment of the selected dataset (1,441 sequences and 4,666 positions) using a Bayesian evolutionary approach based on Markov Chain Monte Carlo (MCMC) implemented in BEAST v.2.6.3 [38], considering a node dating step using the geographic origin as reference metadata. For that, GTR was used because was identified as the best substitution model in the maximum-likehoood initially carried out [39]. The MCMC was then carried out considering a strict clock model and the Bayesian skyline population model, with a chain length of 1,000,000 states and resampling every 10 percent of the states. The sampling was considered as sufficient when the effective sample size (ESS) exceeds 200 for all parameters. The Bayesian skyride analysis was conducted in Tracer v1.7.1 [40]. Tree files were summarized with LogCombiner v1.10.4 (with a burning of 300,000) and then were annotated in Tree Annotator v2.4.8 (with a burning of 5%) (38), with a maximum clade credibility and mean node heights.

### Clinical data and statistical analysis

A Shapiro-Wilk test was performed to assess the normality of age distribution depicted as mean and standard deviation (SD). The categorical variables are shown as frequency proportions with the corresponding 95% Confidence Interval (CI). To assess associations between putative clusters and independent clinical variables (e.g., age, sex, municipality, health care worker status, attention place, close contacts, health status, comorbidities, fever, respiratory symptoms, and ethnicity) an ordinal regression model was performed, and raw and adjusted odds ratios (OR) were calculated. The identified clusters were taken as the dependent categorical variable (0, patient isolates excluded from cluster 1 (C1) and 2 (C2); 1, isolates that comprise C1; 2, isolates that comprise C2). Furthermore, we performed a second analysis to investigate associations between symptomatic patients and distinct clades of each cluster. Multicollinearity diagnostics were performed using variance inflation factor (VIF), tolerance, and eigenvalues [41,42]. The Software STATA14 was used for all the analysis above described, setting the level of significance to 0.05.

### Geographic and statistical analysis

To explore the spatial-temporal dynamics of genomic data from a molecular epidemiologic perspective, we conducted a Topological Data Analysis (TDA) using genomic pairwise distances, coordinates of residence of the individual (manually obtained *in situ*), and the date of each sample (day of symptom onset, if available; day of specimen collection otherwise). We used our own implementation of the Mapper algorithm, following the methodology implemented for other pathogens in previous research [43].

Using the genomic pairwise distance as the similarity measure and the date as the filter parameter, we studied the occurrence and interaction of clusters with similar genomes across time. We then included their geographical relation by constructing the point intersection network and projecting it onto the geographical space. This allowed us to identify central cases happening at key moments and study how they relate geographically.

Although the mapper algorithm is known for having unstable results and parameter selection is crucial for reconstructing the original space, our implementation automatically infers the size and overlap of the filters using a Gaussian Mixture Model over the filter space (the dates of the samples) which removes one metaparameter from the original implementation. Furthermore, using mapper we identified smaller clusters across time, local phenomena that are sometimes missed by analyzing the complete dataset by traditional cluster methods [44,45].

## Results

### Epidemiological analysis

**Fig 1:**
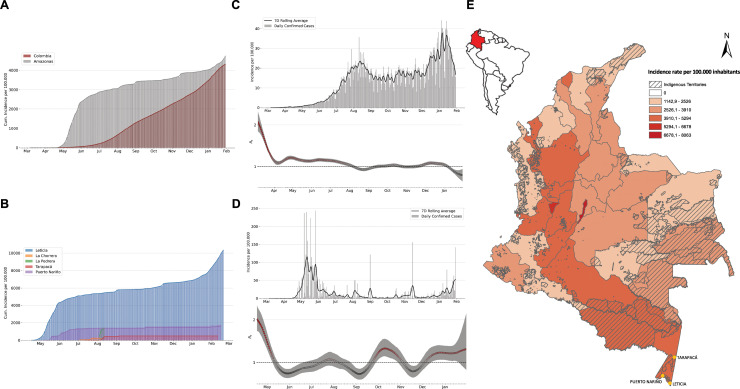
Epidemiological description of SARS-CoV-2 in the Colombian Amazonas department. **A.** Graphical representation of SARS-CoV-2 cumulative incidence in the Amazonas department and Colombia from March to January 30, 2021. The grey bars represent the cumulative incidence per 100.000 people in the Amazonas department and the red bars represent the cumulative incidence per 100.000 people in the rest of the country. **B.** Distribution of the cases in each of the municipalities in the Amazonas department including Leticia, Puerto Nariño, La Pedrera, La Chorrera and Tarapacá. **C.** - **D.** Estimates of the effective reproductive number (with a serial interval of 5.5 and variance of 4.5) where bars in the figure are reported cases in Colombia and the Amazonas department, respectively. The dots in the effective reproductive plot from grey to red show values of this quantity in time above 1 (red dots) and below 1 (grey dots). **E.** Incidence per 100,000 habitants by each Colombian department as of January 30, 2021. The hatched pattern demarcates national indigenous territories, and the yellow dots indicate the municipalities from where specimens were collected (Leticia, Puerto Nariño, and Tarapacá). The map was constructed in QGIS (QGIS Geographic Information System, Open-Source Geospatial Foundation Project, http://qgis.osgeo.org with the layer from the link: https://data-agenciadetierras.opendata.arcgis.com/datasets/fc3fc9592dd8460faf2b7f0bad0f8b33_0.

We estimated and compared the cumulative incidence (per 100,000 inhabitants) of SARS-CoV-2 cases in Colombia and the Colombian Amazonas department, during the period of March 2 to January 30, 2021. We observed an exponential increase of 1.27 to over 4,000 new cases per 100,000 in Amazonas during this period, representing a high proportion of the cumulative incidence across all Colombia (**Fig 1A, S3 Table**). Indeed, a comparison of COVID-19 incidence rates at all territories revealed that Amazonas was one of the most affected; particularly the indigenous territories that include Leticia, Puerto Nariño, and Tarapacá (**Fig 1B**). Estimation of the effective reproductive number (with a serial interval characterized by a Gamma function of mean 5.5 and variance of 4.5) shows that the Amazonas department exhibited important increases in Rt values during four principal moments, in May, at the end of July, in October, and finally, between December and January (**Fig 1C and 1D**). Interestingly, we found that in the Amazonas the outbreaks tend to be more accelerated than in the rest of the country, underscoring the importance of understanding local transmission and contact networks. We also assessed the incidence of SARS-CoV-2 across all other Colombian departments (**Fig 1E**).

The sociodemographic characteristics of the patients were discriminated between indigenous and non-indigenous populations and were summarized in **Table 1**. The mean age was similar between non-indigenous and the indigenous population (45.6 and 48.1 years, respectively), for the non-indigenous population the majority (57.7%) were female, while for the indigenous 85.7% were males. Moreover, all the infections reported by the indigenous population were symptomatic. The majority of patients (N=57) were from Leticia with a minority from Puerto Nariño (N=1) and Tarapacá (N=1) municipalities. Finally, there were not significant differences between the clusters and clades distribution among the ethnicity variable.

**Table 1:** Metadata of the SARS-CoV-2 positive patients (n = 59) sequenced in this study, discriminated by ethnicity.

Variables	Non-Indigenous	Indigenous	p-value
Age, years*	45.6 (18.6)	48.1 (21.7)	
**Sex**			
Female	30 (57.7)	1 (14.3)	0.038
Male	22 (42.3)	6 (85.7)	
**Municipality**			
Leticia	51 (98.1)	6 (85.7)	0.225
outside Leticia^‡^	1 (1.9)	1 (14.3)	
**Health care worker**			
No	49 (94.2)	7 (100)	0.680
Yes	3 (5.8)	-	
**Attention place**			
Home	46 (90.2)	4 (57.1)	0.048
In-Hospital	5 (9.8)	3 (42.9)	
**Close contact**			
No	29 (55.8)	4 (57.1)	0.468
Yes	15 (28.8)	1 (14.3)	
Missing	8 (15.4)	2 (28.6)	
**Comorbidities**			
No	33 (64.7)	4 (57.1)	0.499
Yes	18 (35.3)	3 (42.9)	
**Health status**			
Asymptomatic	9 (17.3)	-	0.293
Symptomatic	43 (82.7)	7 (100)	
**Fever**			
No	20 (38.5)	1 (14.3)	0.207
Yes	32 (61.5)	6 (85.7)	
**Respiratory symptoms**			
No	20 (38.5)	2 (28.6)	0.475
Yes	32 (61.5)	5 (71.4)	
**Clusters**			
Out	21 (40.4)	4 (57.1)	0.375
C1	22 (42.3)	1 (14.3)	
C2	9 (17.3)	2 (28.6)	
**Clade**			
Out	21 (40.4)	4 (57.1)	0.375
G	22 (42.3)	1 (14.3)	
GH	9 (17.3)	2 (28.6)	

The percentages are calculated by columns.

### Phylogenetic analysis of Colombian Amazonas genomes

**Fig 2:**
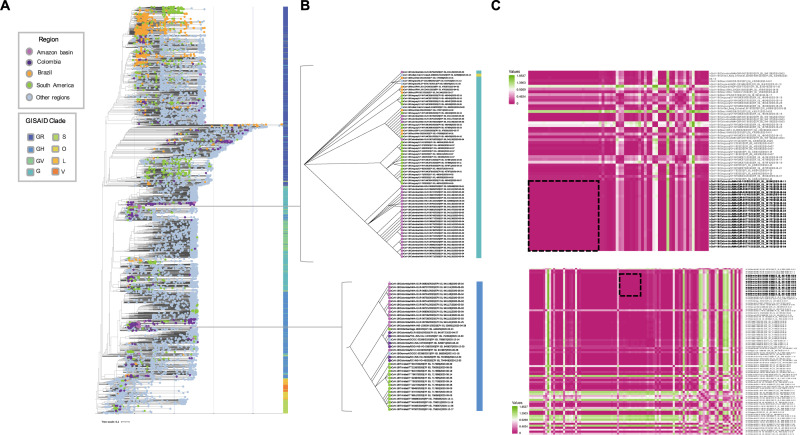
SARS-CoV-2 clusters in the Colombian Amazonas department. **A.** Maximum Likelihood tree built of 9,712 genomes used in this study and worldwide diversity. The colors of the tips indicate the geographical origin of the genomes highlighting the Amazonas department, Colombia, Brazil and South America genomes in specific colors. The colors of the bar on the right indicate the GISAID clade assignment. **B.** Magnification of the two clusters, C1 and C2; isolates colored by region and GISAID clade. **C.** Heatmap visualization of the distance matrix of the clade which include the cluster C1 (up) and the clade which include the cluster C2 (down), the clusters identified in our study are the largest group of Amazonas genomes with the lowest dissimilarity value, C1 and C2, represented by dotted lines within each heatmap.

Maximum likelihood phylogenetic analysis with a sampled global background of 9,653 publicly available genomes, including all South American genomes and representatives from all regions worldwide (until January 30, 2020) was reconstructed. The phylogenetic reconstruction obtained from the complete dataset showed that the 59 Amazonas genomes were distributed between two distinct GISAID clades; Clade G (N = 28 genomes) and Clade GH (N = 31 genomes) (**Fig 2A**). The genomes formed two independent clusters (C1 and C2) principally with other South American genomes, C1 mainly with Uruguay’s genomes and Brazil’s genomes, while C2 with genomes clustered mainly with isolates from other Colombian departments (i.e., Boyaca, Huila, Bolivar, and Tolima), and Trinidad and Tobago’s genomes. Regarding the Pangolin lineages assigned to the Amazonas genomes, we observed the circulation of 10 lineages (B, B.1, B.1.1, B.1.1.237; B.1.1.28, B.1.1.74, B.1.111, B.1.195, B.1.420 and P.1) with an increased number of reports in January, 2021 (**S1 and S2 Tables; S1 Fig)**.

**Fig 3:**
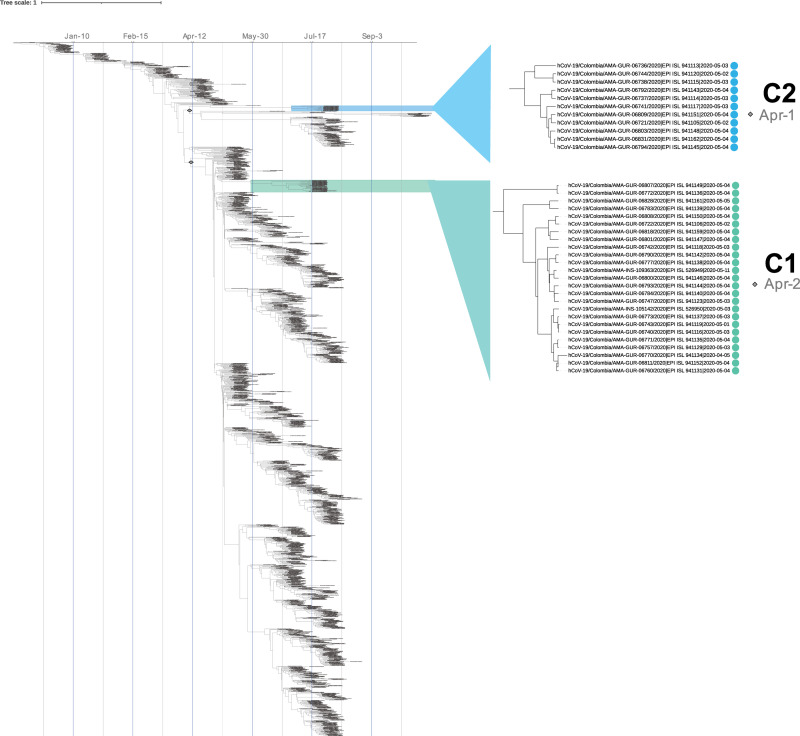
Bayesian phylogenetic tree built from 1,436 genomes including the 59 Colombian Amazonas sequences of this study and a worldwide representation. Both clusters identified in this study are highlighted in specific colors. The color cyan represent the cluster 1 and the GISAID clade G, and the blue color represent the cluster 2 and the GISAID clade GH. A zoom showing the IDs of the genomes belonging to each cluster is showed on the right panel. Additionally, the diamond figures represent the putative introduction dates, April 1 and April 2, 2020, respectively.

We utilized maximum likelihood phylodynamic analyses to infer transmission events between Amazonas cases. Posteriorly, the clusters with the Amazonas genomes were pruned (**Fig 2B**) and a distance matrix was calculated between genomes of each cluster to identify clusters of related genomes (**Fig 2C**). In the C1, we observed a cluster of 23 Amazonas genomes of this study grouped with 2 Amazonas genomes downloaded from GISAID, for a total of 25 highly similar genomes. These presented a moderate to high dissimilarity to the remaining genomes in the cluster **(Figs 2B, 2C and 3)**. Analogously, we identified another cluster of 11 Amazonas cases within the C2, based on the high degree of similarity between them and notably differences with the remaining genomes **(Figs 2B, 2C and 3)**. For each cluster, multiple single nucleotide variants (SNVs) distinguished the Amazonas isolates from other closely related genomes. Genomes within C1 were uniquely characterized by four substitutions in the ORF1ab (C3037T, T4213C, A6466G, and C14408T), and 1 substitution in the S gene (A22110G) (**S4 Table**). Similarly, isolates within the C2 cluster shared four mutations in the ORF1ab (C3037T, C10507T, C14408T, and C18877T), and one in the S gene (G25563T) (**S4 Table**). Of note, the widely reported D614G substitution [46] in the S gene was present in all Amazonas isolates.

Interestingly, the C1 predominantly clustered with Uruguayan and Brazilian genomes while those in the C2 were mainly associated with genomes from Colombia and Trinidad and Tobago **(Fig 2B and 2C**). In addition to the maximum-likelihood analysis, we performed a Bayesian phylogenetic analysis-based on a divergence dating with sampled ancestors (**Fig 3**). In both phylogenetic analysis the same tree topology was observed, with two clusters grouping the majority of the Amazonas genomes evaluated in this study. In addition, the inferred time to the most recent common ancestor (tMCRA) was obtained from the Bayesian phylogeny due to the robustness of this kind of analysis; for the pruned clade containing C1 was April 2, 2020, this cluster was closely related to Brazilian and Uruguayan genomes suggesting an independent introduction to the region from the South of the continent. On the other hand, the putative introduction date of the clade that included C2 was April 1, 2020. As we described earlier this cluster was associated mainly to other Colombian genomes, thus, these results suggest that the C2 outbreak could be result from a local introduction of SARS-CoV-2 into the Colombian Amazonas department. Additionally, the C2 was also closely related to Trinidad and Tobago´s genomes, country separated from Colombia by the Caribbean sea, representing a major geography dispersion of the genomes grouped in this cluster, furthermore, the dates of these isolates had a bigger temporal window (between March, 2020 to January, 2021), thus the members were dispersed widely in a bigger spatial-temporal scale, probably as a consequence of the lifting of the lockdowns and the reopening of the flights in different countries.

We next used logistic regression analyses to assess the sociodemographic and clinical characteristics of each cluster (C1 and C2) compared to non-outbreak cases (**Table 2**). Nevertheless, the logistic regression analysis did not show significative associations between the clusters and the demographic/clinical characteristics **(Table 2)**. The second regression model allowed to estimate the association between the symptomatic patient status and the cluster or the GISAID clade identified **(Table 3)**. The results showed negative associations (OR: 0.09; 95% CI 0.01-0.82) with C1, and the clade G (OR: 0.07; 95% CI 0.01-0.98) **(Table 3)**.

**Table 2:** Ordinal logistic regression model to assess the association between sociodemographic variables and transmission chain. C1 (N = 23); C2 (N = 11); Out, represent the remaining Amazonas genomes (N = 25) evaluated in this study.

	CLUSTER			UNIVARIATE ANALYSIS		MULTIVARIATE ANALYSIS	
Variables	Out	C1	C2	*OR*	*95%CI*	aOR^*^	*95%CI*
**Increasing age**	-	-	-	10.98	0-96-1.01	0.90	0.95-1.03
	*n (%)*	*n (%)*	*n (%)*				
**Sex**							
*Female*	13 (52.0)	12 (52.2)	6 (54.5)	Reference		Reference	
*Male*	12 (48.0)	11 (47.8)	5 (45.5)	0.94	0.36-2.45	1.31	0.36-4.72
**Municipality**							
*Leticia*	24 (96.0)	22 (95.6)	11 (100)	Reference		Reference	
*Outside Leticia*^*†*^	1 (4.0)	1 (4.4)	-	0.56	0.42-7.54	1.83	0.05-6.40
**Indigenous ethnicity**							
No	21 (84.0)	22 (95.6)	9 (81.8)	Reference		Reference	
Yes	4 (16.0)	1 (4.4)	2 (18.2)	0.74	0.14-3.74	0.20	0.01-3.56
**Health care worker**							
*No*	24 (96.0)	22 (95.6)	10 (90.9)	Reference		Reference	
*Yes*	1 (4.0)	1 (4.4)	1 (9.0)	1.84	0.20-6.97	0.85	0.04-7.37
**Attention place**							
*Home*	20 (83.3)	20 (87.0)	10 (90.9)	Reference		Reference	
*In-Hospital*	4 1(16.7)	3 (13.0)	1 (9.1)	0.64	0.15-2.65	1.07	0.10-10.77
**Close contact**							
*No*	16 (72.7)	12 (60.0)	5(71.4)	Reference		Reference	
*Yes*	6 (27.7)	8 (40.0)	2 (28.6)	1.31	0.43-4.02	1.10	0.28-4.38
**Health status**							
*Asymptomatic*	2 (8.0)	7 (30.4)	-	Reference		Reference	
*Symptomatic*	23 (92.0)	16 (69.6)	11 (100)	0.82	0.24-2.74	1.86	0.11-5.17
**Comorbidities**							
No	15 (62.5)	15 (65.2)	7 (63.6)	Reference		Reference	
Yes	9 (37.5)	8 (34.8)	4 (36.4)	0.93	0.34-2.55	1.70	0.43-6.70
**Fever**							
*No*	6 (24.0)	12 (52.2)	3 (27.3)	Reference		Reference	
*Yes*	19 (76.0)	11 (47.8)	8 (72.7)	0.63	0.23-1.68	0.46	0.06-3.31
**Respiratory symptoms**							
*No*	6 (24.0)	12 (52.2)	4 (36.4)	Reference		Reference	
*Yes*	19 (76.0)	11 (47.8)	7 (63.4)	0.51	0.19-1.38	0.42	0.09-1.91

^*†*^Outside Leticia: correspond to Puerto Nariño and Tarapacá.

*Estimates of logistic regression were adjusted for (OR adjusted): age, sex, municipality, ethnicity, health care worker, attention place, close contact, health status, comorbidities, fever and respiratory symptoms.

Values in bold p ≤ 0.05.

aOR= odds ratio adjusted; 95%CI= 95% confidence interval.

**Table 3:** Ordinal logistic regression model to assess the association between clusters, GISAID clade and patient status. C1 (N = 23); C2 (N = 11); Out, represent the remaining Amazonas genomes (N = 25).

	STATUS		UNIVARIATE ANALYSIS		MULTIVARIATE ANALYSIS	
	*Asymptomatic*	*Symptomatic*	*OR*	*95%CI*	aOR^*^	*95%CI*
**Cluster**						
Out	2 (22.2)	23 (46.0)	Reference		Reference	
C1	7 (77.8)	16 (32.0)	0.19	0.05-1.44	**0.09**	**0.01-0.82**
C2	0	11 (22.0)	1.03	0.88-1.97	3.10	0.68-6.93
**Clade**						
Out	2 (22.2)	23 (46.0)	Reference		Reference	
*G*	7 (77.8)	16 (32.0)	0.19	0.03-1.08	**0.07**	**0.01-0.98**
*GH*	0	11 (22.0)	0.93	0.88-2.08	2.08	0.98-4.57

*Estimates of logistic regression were adjusted for (OR adjusted): age, sex, municipality, ethnicity, health care worker, attention place, close contact, health status and comorbidities.

Values in bold p ≤ 0.05.

aOR= odds ratio adjusted; 95%CI= 95% confidence interval.

### Spatial-temporal transmission analyses

**Fig 4:**
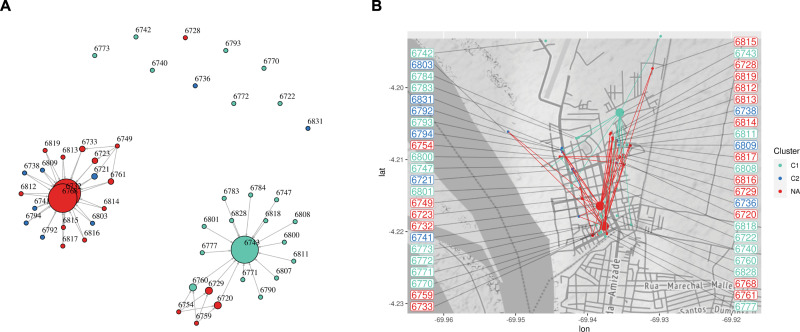
SARS-CoV-2 Spatial-temporal analyses A. Point intersection network of interconnected clusters of isolates within distinct clusters. Size of vertices are proportional to number of isolates. Color of points reflect clusters that correspond with C1 (cyan), C2 (blue) or not (red). **B.** Spatial projection of the point intersection network over the streets of Leticia, Amazonas.

To further characterize SARS-CoV-2 transmission across Amazonas department, we performed a topological data analysis using genomic pairwise distances and geographical data of the sequenced isolates (**Figs 4 and S2**). This figure can be understood as clusters of cases (by genetic distance), where each single case is pointing towards the most central node (case) in their cluster. Connections represent overlapping genetic clusters across time intervals, and therefore the image can be constructed as a geographical representation of the relative frequencies over time.

Using the *Mapper* algorithm as previously described [43,47] we observed two predominant transmission networks, depicted as two large connected components in the point intersection network [43] with one and two central cases respectively **(Fig 4A)**. Although ten isolates did not connect to these two main components, this can be attributed to small date gaps (3-4 days without cases) in the filter. Projection of the point intersection network over the street map of Leticia, Colombia revealed overlap of the clusters (**Fig 4B**). However, the central nodes of each network are located at opposite ends of the city suggesting distinct epidemiological dynamics of SARS-CoV-2 transmission within Leticia. Our data also indicate that during the outbreak the cases in both clusters increased with a similar frequency, suggesting that the clusters represent two independent transmission events.

## Discussion

As of February 28, 2021, the Americas are among the most affected regions in the world. United States, Brazil, and Colombia, are among the top nations worldwide with the highest number of cases [47]. This highlights the COVID-19 burden in South America and the utility of genomic surveillance to decipher transmission events in order to address further spread in such regions characterized by poverty, limited access to healthcare resources, and overwhelmed or absent infrastructures.

While limited studies have investigated the distinct SARS-CoV-2 transmission events in South America [24,25,48–54], only one has utilized genomic data to characterize viral spread in the Amazon basin region in Brazil [53]. We present the first study of SARS-CoV-2 spread within the Colombian Amazon basin region, whose communities are culturally and ethnically rich, but are particularly susceptible to infectious diseases [10–13]. Indeed, despite mandatory lockdowns and limited geographic access to the region, the first case of SARS-CoV-2 in the Colombian Amazonas department was confirmed on April 7 [9] which quickly spread to such an extent that Amazonas continue to be one of the departments with the highest incidence in almost one year of the public health emergency (**Fig 1**). This rapid and continue spread could be attributed to different social and cultural variables of the region such as a limited adherence to the lockdown conditions, the reduced access to information and/or a reduced ability to social distance.

We sequenced 59 Amazonas patient isolates and identified two distinct clusters, whose arrivals date back to early April 2020. Clustering of the virus isolates with either Brazilian/Uruguayan genomes, or with Colombian/Trinidad and Tobago genomes, suggests that SARS-CoV-2 introductions into the region likely stem from multiple distinct events (**Fig 2**) and have different transmission dynamics, one more restrained spatiotemporally and the other with a bigger spread along the months and even related with isolates form Trinidad and Tobago, country separated by the Caribbean sea; this highlighting the great impact that the different governmental measurements have in the transmission of the virus across regions, such as the lifting of the lockdowns and the reopening of national and international flights.

The transmission dynamic in the Colombian Amazonas department is possibly related to a first event where infected Colombian citizens brought the virus prior to lockdowns or travel bans concurrent with an introduction via traffic at the Colombia-Brazil Amazon border (e.g., at Tabatinga, Brazil and Leticia, Colombia). Furthermore, this suggests that untracked SARS-CoV-2 community transmission occurred prior to the first confirmed case (April 7, 2020) **(Fig 3)**. Such transmission dynamics has been reported in other countries like the US (e.g., Washington State [20], California [21]) and Panama [26] where viral circulation was reported weeks prior to the first confirmed cases.

Interestingly, all isolates in this study encode the D614G spike variant which has been associated with higher viral loads and increased infectivity and transmissibility [46,55]. Moreover, both clusters, C1 and C2, presented changes in the ORF1ab and the S gene, and while concurrent mutations in the spike protein have been reported to alter infectivity of the D614G variant, the impact of the other substitutions have been assessed in a minor proportion, however, these with the substitutions in other genome regions, such as ORF1ab, are critical for viral replication including replication of the RNA genome, packaging of budding virions, viral transcription, suppression of host immune responses, and suppression of host gene expression [56,57]. Further studies are required to determine the consequences of these viral mutations.

Early tracing studies demonstrated that many infections and clusters are driven by sustained contact and group activities [18,58–61], and further analyses that incorporate contact and movement data can provide additional insight. Multiple clusters of SARS-CoV-2 infections have been reported in association with family, religious, and work gatherings and group activities [62–65]. The two transmission clusters in the Colombian Amazonas department reported herein are potentially the result of group activities and/or settings in Leticia. Both these clusters were situated in a 5 km^2^ area with an extensive interactive network of 51 bars and restaurants, 34 hotels and hostels, and 4 churches (**Fig 4**). Moreover, these results highlight the more local insight that the TDA analysis could contribute to track the transmission dynamics of small clusters over the geographical space. In this regard, the Bayesian phylogenomic reconstruction supported two clear clades **(Fig 3)** that were validated by spatial-temporal analyses **(Fig 4)**. In the TDA analyses, only C1 could be clearly validated that could be explained by the low number of genomes found in C2 or even gaps in the data. Nevertheless, both analyses **(Figs 3 and 4)** give us information of the relatedness of isolates (in the case of C1) which highlights the utility of combining Bayesian phylogenomic studies and TDA. Future studies should sequence additional samples in concurrent spatial and temporal scales to fully understand the complex landscape of the SARS-CoV-2 transmission in Leticia, Amazonas.

Although time and space are key variables in characterizing viral spread, symptomatology of those infected also influences transmission dynamics. Despite we did not find significant differences between the indigenous and non-indigenous population among the demographic/clinical variables, the regression analysis suggests that in Leticia, transmission of the C1 and the G clade isolates were associated with symptomatic infection (**Tables 2 and 3**). This could reflect a transmission process occurring in the Amazonas department, driven by asymptomatic hosts whose viruses are closely related to Brazilian/Uruguayan isolates. Interestingly, several studies have demonstrated a reduced risk of spread for asymptomatic versus symptomatic cases [66–68]; we demonstrate, however, that viral transmission in the Amazonas could be occurring from asymptomatic infections principally. However, our study would benefit greatly from improved sampling at the arrival of SARS-CoV-2 and through its continued spread to best resolve these epidemiologic events. Nonetheless, the sample size, the quantity of covariables and the number of indigenous genomes included in this study, principally due to the limited self-recognition as indigenous and the lack of adequate registration of these communities in the formats of new SARS-CoV-2 cases notification authorized; and used along the country could affect the significance of the statistical estimations. For future studies in indigenous communities, the adequate collection of these variables should be improved by national and governmental authorities.

In addition to the impacts of the dynamics described above, we identified the circulation of ten lineages in the department including the P.1 lineage (VOC) **(S1 Fig)**. Also, all the genomes with the predominant DG614 mutation. It is crucial to evaluate the possible transmission and epidemiological consequences that the new VOCs circulating around the world could generate, especially the VOC P.1, variant briefly reported in the first trimester of the 2021 in the Brazilian territory despite the high seroprevalence reported in October 2020 (76%) [15,69,70], suggesting that this second wave in Brazil is the result of the dissemination of the P.1 variant which is related to a major transmissibility, a major viral load and possible events of reinfection [15,17]. This P.1 is already circulating in the Colombian Amazonas department and in the recent weeks an increase in the number of COVID-19 cases has been observed. There is an urgent need to improve the genomic surveillance of SARS-CoV-2 in this department, evaluating the pandemic dynamics and most importantly the implementation and efficacy of the national vaccination program in the light of P.1 circulation.

Despite the difficulty in the identification of the members of the different indigenous communities due to the lack of registration and notification of these individuals, our study is the first to shed light on distinct SARS-CoV-2 introductions and outbreaks in the territory where the most indigenous communities inhabit in Colombia. These findings enable implementation of strategies to target COVID-19 as previously described [8,59–73]. More importantly, these data provide insight into unique SARS-CoV-2 infection dynamics in a region whose inhabitants are particularly vulnerable to infectious disease due to both extrinsic (e.g., socioeconomic, limited government and medical infrastructures, among others) and intrinsic (e.g., genetic homozygosity) factors [8,11]. Importantly, given the rich ethnic and cultural diversity of these communities and the circulating of a new variant of concern (P.1), these aspects highlight the importance of genomic surveillance for effective tracking of the SARS-CoV-2 evolution and the implementation of proper prevention and control measures to protect these individuals.

## Supporting information

S1 Fig Bar plot with the distribution of the ten Pangolin lineages detected in the Amazonas genomes in the different months of sampling. (PDF)

S2 Fig SARS-CoV-2 Mapper 1-skeleton A. Topological depiction of clustered SARS-CoV-2 isolates in a 1-skeleton diagram. Vertices on flares reflect clusters of isolates and the size of vertices reflect the number of isolates within each given cluster. **B.** Temporal projection of the 1-skeleton as a function of days after April 5, 2020. Nodes are uniformly arranged on the y axis to avoid overlap and visualize the possible flares.
(PDF)

S1 Table Metadata of the genomes retrieved from GISAID and employed in the maximum-likelihood phylodynamic analysis in TreeTime. (XLSX)

S2 Table Subsampled of the global background diversity genomes retrieved from GISAID and employed in the Bayesian phylodynamic analysis in BEAST. (XLSX)

S3 Table Data use for the calculation of the Amazonas and Colombia incidence. (XLSX)

S4 Table Substitutions presented in each of the clusters identified in the study, C1 and C2. (PDF)
