# Supplementary figures and images for: Deciphering the introduction and transmission of SARS-CoV-2 in the Colombian Amazon Basin
Source: PLoS Negl Trop Dis. 2021 Apr 15;15(4):e0009327. doi: 10.1371/journal.pntd.0009327 (PMC8078805; doi:10.1371/journal.pntd.0009327)

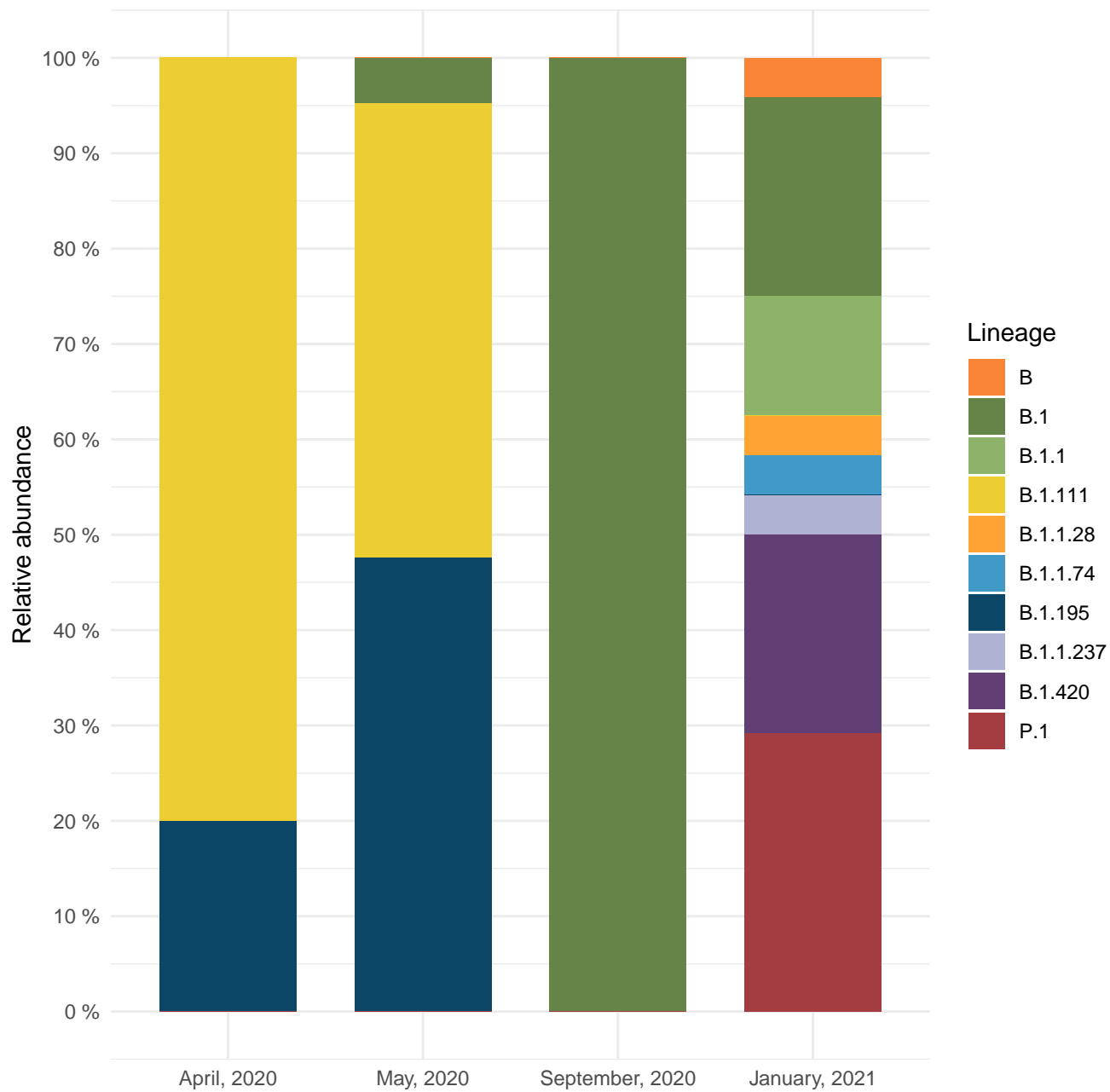

Supplement: S1 Fig — (PDF) [file pntd.0009327.s001.pdf]

A

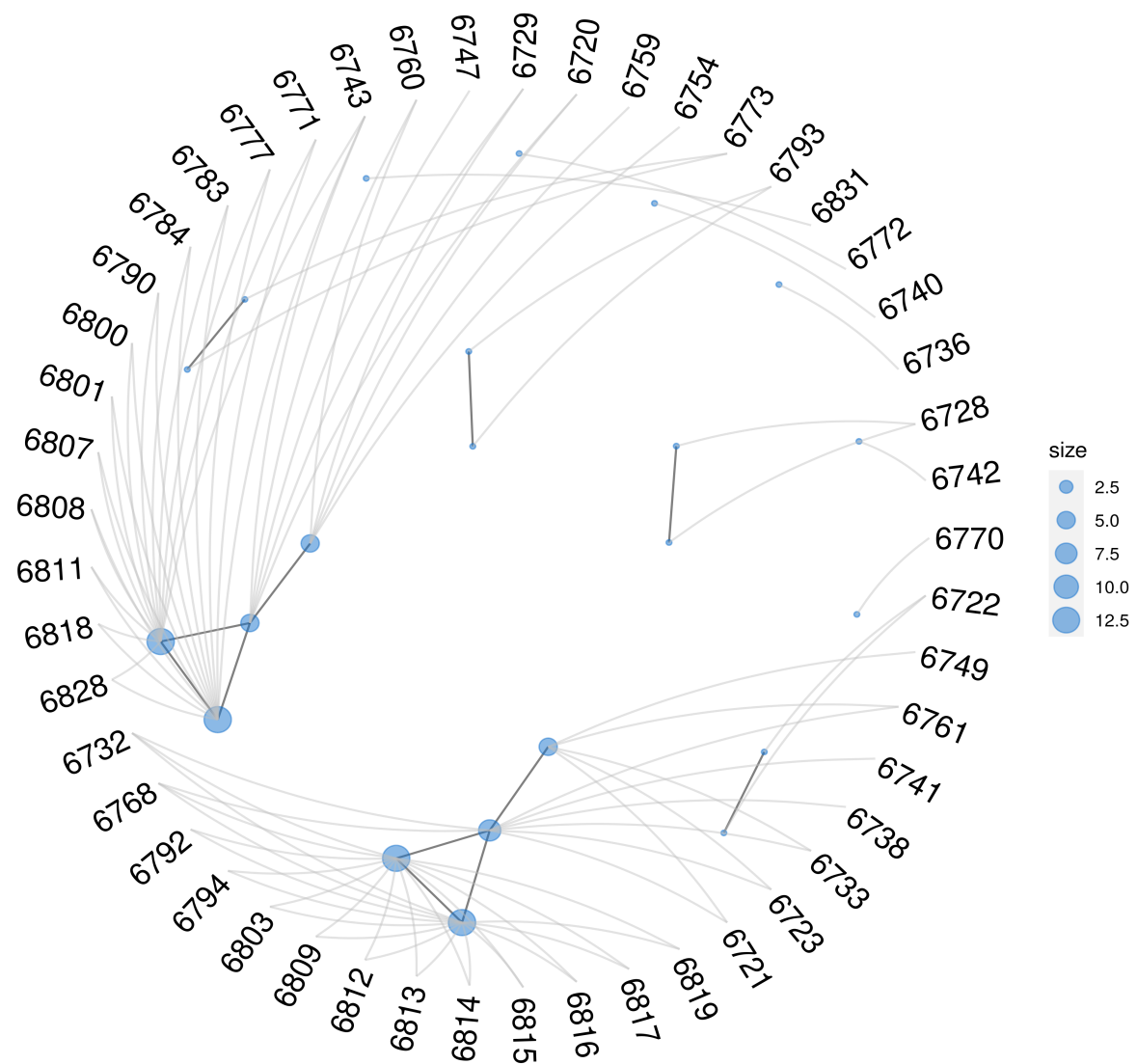

B

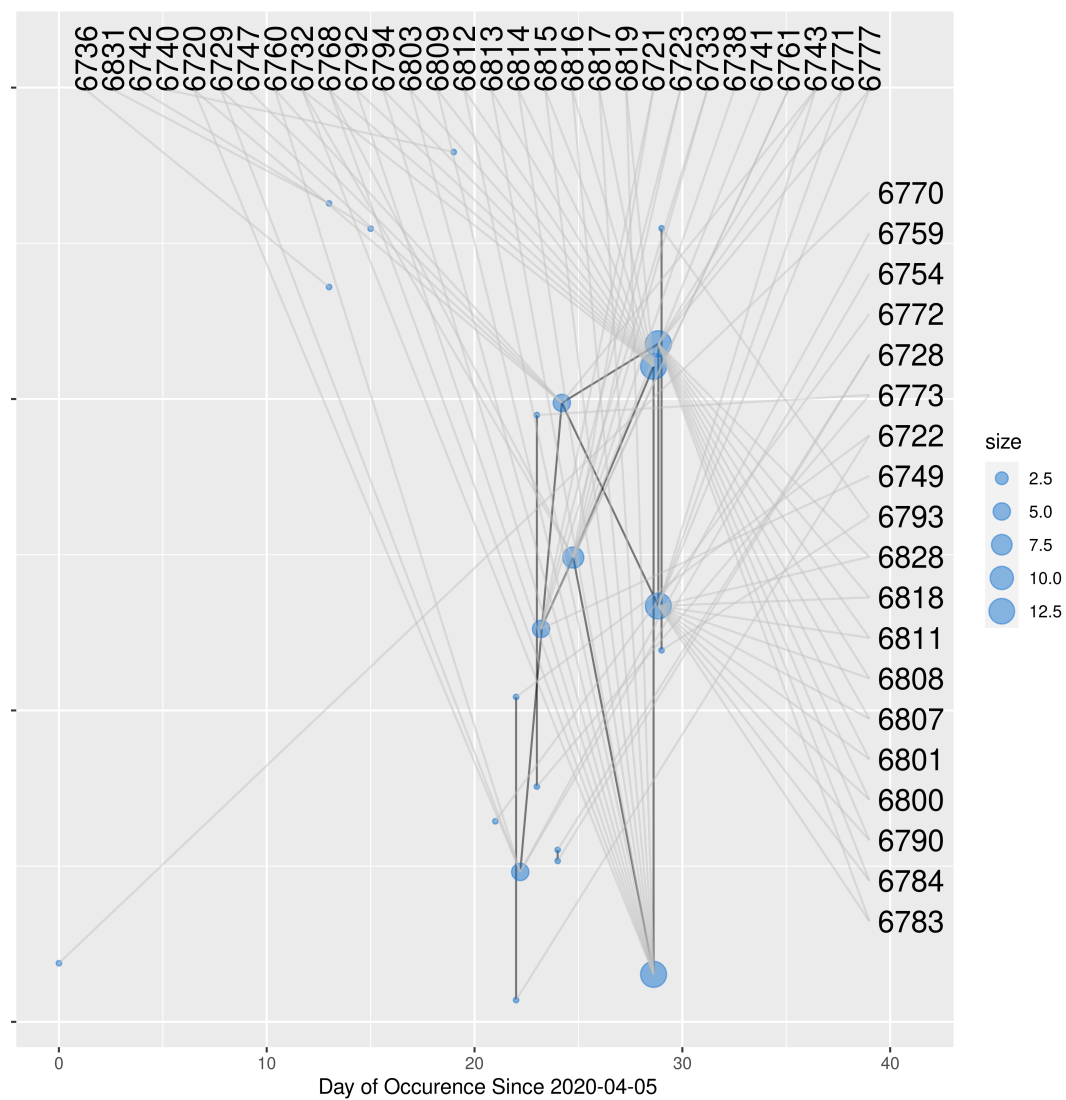

Supplement: S2 Fig — Topological depiction of clustered SARS-CoV-2 isolates in a 1-skeleton diagram. Vertices on flares reflect clusters of isolates and the size of vertices reflect the number of isolates within each given cluster. B. Temporal projection of the 1-skeleton as a function of days after April 5, 2020. Nodes are uniformly arranged on the y axis to avoid overlap and visualize the possible flares. (PDF) [file pntd.0009327.s002.pdf]
