# Supplementary material for: Deciphering the introduction and transmission of SARS-CoV-2 in the Colombian Amazon Basin
Source: PLoS Negl Trop Dis. 2021 Apr 15;15(4):e0009327. doi: 10.1371/journal.pntd.0009327 (PMC8078805; doi:10.1371/journal.pntd.0009327)
Supplement: S4 Table — (PDF) [file pntd.0009327.s006.pdf]

Supplementary Table 4. Substitutions presented in each of the clusters identified in the study, C1 and C2.

Cluster	Substitution	Number of Sequences	ORF
C1	C3037T	25	ORF1ab
	T4213C	25	ORF1ab
	A6466G	25	ORF1ab
	C14408T	25	ORF1ab
	A22110G	25	S gene
	A23403G	25	S gene
C2	C3037T	11	ORF1ab
	C10507T	11	ORF1ab
	C14408T	11	ORF1ab
	C18877T	11	ORF1ab
	A23403G	11	S gene
	G25563T	11	S gene
